# Evaluation of the Analgesic Effect of Adding Neostigmine to Lidocaine in Intravenous Regional Anesthesia for Carpal Tunnel Syndrome Surgery

**DOI:** 10.7759/cureus.87010

**Published:** 2025-06-29

**Authors:** Jasim M Salman, Huda M Hadi

**Affiliations:** 1 Anesthesiology and Critical Care, University of Basrah, College of Medicine, Basrah, IRQ; 2 Anesthesiology, Basrah Health Directorate, Basrah, IRQ

**Keywords:** adjuncts, carpal tunnel syndrome, intravenous regional anaesthesia, lidocaine, neostigmine

## Abstract

Background: Neostigmine, a reversible acetylcholinesterase inhibitor (AChEI), has shown potential as an adjunct to local anesthetics like lidocaine in peripheral nerve blocks, including carpal tunnel syndrome (CTS). It may prolong analgesia by activating muscarinic receptors involved in pain modulation. Some studies report that adding neostigmine to lidocaine improves the duration of anesthesia and postoperative pain relief, particularly in upper limb procedures. However, results are mixed, possibly due to differences in doses or nerve barrier permeability. Overall, neostigmine’s role in enhancing analgesia in nerve blocks such as Bier's block and CTS remains an active area of investigation.

Aim of study: This study aims to assess the analgesic efficacy of adding 0.5 mg neostigmine to 2% lidocaine in adult patients undergoing upper limb surgery under intravenous regional anesthesia (IVRA). Specifically, it evaluates the impact of this combination on intraoperative and postoperative pain, analgesic requirements, and recovery profiles, thereby contributing to improved perioperative pain management strategies.

Patients and methods: A total of 52 patients admitted to Al-Fayhaa Teaching Hospital, Basrah, Iraq, were randomized into two groups of 26 patients each. One patient excluded from analysis from each group. In the control group, local anesthesia of 3 mg/kg lidocaine was administered with 40 mL of normal saline. While the neostigmine group patients received 3 mg/kg lidocaine with 0.5 mg neostigmine, the same amount of saline was administered. Physiological parameters, sensation, and motor activity onset time, and recovery time after intravenous regional anesthesia were registered.

Results: The neostigmine group included males (4%) and females (96%) with a mean age of 41.76±5.69 years, while the control group included males (20%) and females (80%) with a mean age of 37.6±5.00 years. There were no differences in the demographic data (American Society of Anesthesiologists (ASA), gender, weight), in addition to pinprick onset and recovery times, touch onset time, and block recovery time between both groups. A significant difference was observed in the age, surgical duration time, tourniquet time, touch recovery time, and motor block onset time between both groups (p < 0.05). In addition, no significant differences in postoperative complications were observed between the two groups (p = 0.074). However, there was a significant association in analgesic need, whether intraoperative or to reduce tourniquet pain, among the compared groups (p < 0.001).

Conclusion: The addition of neostigmine to lidocaine in the surgical treatment of CTS shows no significant benefits regarding postoperative pain relief, but it is of benefit during the operation.

## Introduction

Carpal tunnel syndrome (CTS) is a symptomatic compression neuropathy of the median nerve in the wrist, affecting approximately 3.8% of the population. Surgical treatment often employs regional anesthesia techniques, including intravenous regional anesthesia (IVRA) [1-3].

Introduced by Karl August Bier in 1908, IVRA involves injecting a local anesthetic intravenously after occluding venous drainage with a pneumatic tourniquet. This technique is simple, effective, and boasts a success rate of 96% to 100% [4, 5]. Contraindications include difficulty in obtaining IV access and certain medical conditions [6]. Despite its safety, potential complications such as nerve damage and tourniquet pain may arise [7]. Patients receiving IVRA generally have quicker recovery times and lower demands for analgesia compared to general anesthesia [8].

An effective IVRA solution should achieve rapid onset, prolonged analgesia, and minimal side effects [9]. Lidocaine, a widely used local anesthetic since its introduction in the 1940s, is favored for its safety but can have side effects at high plasma concentrations [10, 11]. To enhance IVRA, adjuncts like neostigmine are being explored [12].

Neostigmine, an acetylcholinesterase inhibitor (AChEI), can potentiate analgesia by preventing the breakdown of acetylcholine, affecting both central and peripheral ACh receptors [13, 14]. Recent research explores the use of neostigmine as an adjunct to lidocaine to improve analgesic outcomes. However, a direct comparison of the efficacy of lidocaine alone versus lidocaine with neostigmine in IVRA is lacking, which this study aims to address. Furthermore, neostigmine is cost-effective and remains the most common agent used for reversing neuromuscular blockade [15-17].

This study aims to assess the analgesic efficacy of adding 0.5 mg neostigmine to 2% lidocaine in adult patients undergoing upper limb surgery under IVRA. Specifically, it evaluates the impact of this combination on intraoperative and postoperative pain, analgesic requirements, and recovery profiles, thereby contributing to improved perioperative pain management strategies.

## Materials and methods

This study was granted approval by the Iraqi Council for Medical Specialization and the Scientific Council for Anesthesia and Intensive Care. The study was approved under reference number AIC 1/2019 dated 30/10/2019. The study was conducted in accordance with the Consolidated Standards of Reporting Trials (CONSORT) 2010 guidelines to ensure transparent and standardized reporting of this randomized controlled trial. It was carried out at the Department of Anesthesiology, Al-Fayhaa Teaching Hospital, Basrah, in southern Iraq, and was implemented between November 2019 and December 2021.

Based on a preliminary pilot study and using standard sample size calculation for comparison of two means, the following parameters were considered: a power of 90% (β = 0.10), a significance level of 0.05 (α = 0.05), a standard deviation of 11 minutes, and a minimum clinically significant difference of 10 minutes in surgical time.

Therefore, the calculated sample size was approximately 26 patients per group (total of 52 participants) to detect a clinically meaningful difference with adequate power and statistical significance. Two patients were excluded as they didn't meet the criteria. 

Fifty adult patients, American Society of Anesthesiologists (ASA) grades I or II, between 18 and 65 years of age, who presented for elective surgery of the upper limb (CTS), were enrolled. Inclusion criteria required informed consent by the participants, whereas exclusion criteria included regional anesthesia contraindications (e.g., infection at the site of injection or coagulopathy), allergy to neostigmine or lidocaine, and presence of major cardiac, respiratory, neurological, renal, or other systemic disease, or pregnancy or lactation.

All participants gave written informed consent. The patients were randomly categorized using computer-generated randomization into two groups of 26 each, and allocation concealment was achieved through sealed opaque envelopes opened just prior to intervention. The study was conducted in a double-blind manner, with both the patients and the assessors unaware of group assignments. Sensory and motor evaluations were performed by trained, blinded assessors using standardized procedures. The timing of onset and recovery of sensory and motor blocks was recorded by the same blinded personnel at predetermined intervals. Data collection was documented systematically on case report forms and later entered into a secure database. The control group, Group A, was administered 3 mg/kg of lidocaine diluted to 40 ml, while the experimental group, Group B, was administered the same dosage of lidocaine with the addition of 0.5 mg of neostigmine, diluted to 40 ml. The dose of 0.5 mg of neostigmine was selected based on prior studies demonstrating safety and efficacy at this level [18]. Two cannulae of size 20 were utilized for the procedure, one on the dorsum of the operating hand for administration of local anesthetic solution and the other on the opposite arm for intravenous solutions and additional drug administration.

After the exsanguination by the elevation of the arm for two to three minutes using an Esmarch bandage, a double pneumatic tourniquet was then used and inflated to 300 mmHg proximally, with circulation guaranteed to be severed by the absence of palpable peripheral pulses prior to drug injection. The sensory and motor evaluation comprised both the surgical and anesthetic examination. Sensory blockade was quantified with a pinprick test on a 22-gauge short beveled needle every 30 seconds following injection, and motor blockade was quantified by patient finger and wrist movement. Onset time to achieve sensory and motor blockade was determined as the interval from drug administration until there was sensory block in all dermatomes and voluntary motor movement was lost. 

Once proper sensory and motor blockades were achieved, the distal tourniquet was maintained at 300 mmHg while deflating the proximal cuff, and the operation started. Monitoring of all the vital parameters, like mean arterial pressure, heart rate, and oxygen saturation, was carried out prior to inflation of tourniquets and upon release during the operation. Sedative (midazolam, 0.02-0.05 mg/kg) and analgesic (ketamine, 0.1-0.3 mg/kg) requirements were also noted.

Postoperatively, the tourniquet was released when the surgeon finished the last stitch in a cyclic mode, and recovery times were measured: recovery time for sensation was from tourniquet release to the restoration of feeling in all dermatomes, and motor recovery time was from release until the restoration of voluntary movement of the fingers. 

Pain was assessed using the Visual Analog Scale (VAS). Patients’ pain levels were evaluated at specified intervals, and additional analgesics were administered based on the recorded pain scores. The cutoff point for initiating further analgesic intervention was a VAS score of ≥4. The type of analgesics used for breakthrough pain included 1,000 mg intravenous paracetamol. Further, the time to the first request for analgesia was noted. Untoward effects of nausea, vomiting, rash, tachycardia, bradycardia, hypotension, hypertension, dizziness, tinnitus, and hypoxia were noted in patients who were observed in the recovery room to ensure patient safety.

Statistical comparison involved presenting the data in terms of means with standard deviations or percentages. For comparing categorical outcomes, such as the incidence of side effects, the chi-square test was used. For continuous or ordinal data, such as the duration of recovery, the independent samples t-test was used if the data were normally distributed. A p-value of less than 0.05 was considered statistically significant.

## Results

A total of 52 patients were enrolled, with one patient excluded from analysis from each group, leaving 50 participants divided into two groups of 25 patients each. The study group included 4% males and 96% females, with a mean age of 41.76±5.69 years, while the control study group included 20% males and 80% females, with a mean age of 37.6±5.00 years. There were no differences in the demographic data (gender, weight, ASA class) between both groups (Table 1), where values are presented as means ± SD or number (percentage). P-values are based on t-tests for continuous variables and chi-square tests for categorical variables.

**Table 1:** Demographic and surgical data ^a ^t-test was used for continuous variables; ^b^ chi-square test was used for categorical variables; ASA: American Society of Anesthesiologists; min: minutes

Variables	Controls group	Study group	P-value
Age (years, mean ± SD)	37.6 ± 5.00	41.76 ± 5.69	0.009^a^
Gender: male, n (%)	5 (20.0%)	1 (4.0%)	0.082^b^
Gender: female, n (%)	20 (80.0%)	24 (96.0%)	0.082^b^
Weight (kg, mean ± SD)	75.20 ± 10.78	74.88 ± 8.48	0.908^a^
ASA I, n (%)	3 (12%)	0 (0%)	0.07^b^
ASA II, n (%)	22 (88%)	25 (100%)	0.07^b^
Surgical duration (min, mean ± SD)	37.40 ± 11.09	31.20 ± 5.53	0.016a
Tourniquet time (min, mean ± SD)	39.20 ± 6.21	49.76 ± 6.55	<0.001a

This table presents a comparison of demographic and perioperative characteristics between the control group and the study group. Variables include age (mean ± standard deviation), gender distribution (number and percentage), weight (mean ± standard deviation), ASA classification (number and percentage for each class), surgical duration (minutes), and tourniquet time (minutes). The P-values assess the statistical significance of differences between groups, with values less than 0.05 indicating statistically significant differences. Specific P-values are provided for each comparison, and significance levels are denoted accordingly.

Also, there was no significant difference in the pinprick onset time, touch onset time, pinprick recovery time, and motor block recovery time between the two groups (Table 2).

**Table 2:** Onset and recovery from sensory and motor block (min) ^a ^t-test was used for all comparisons; min: minutes

Variables	Controls group	Study group	P-value^a^
Touch onset time (min)	7.12 ± 1.201	7.32 ± 1.069	0.537
Pinprick onset time (min)	7.52 ± 1.327	6.88 ± 1.166	0.076
Motor block onset time (min)	13.64 ± 1.350	14.80 ± 0.957	0.001
Touch recovery time (min)	3.48 ± 0.872	4.16 ± 0.746	0.005
Pinprick recovery time (min)	4.56 ± 1.356	4.80 ± 0.913	0.467
Motor block recovery time (min)	2.88 ± 0.781	2.68 ± 0.852	0.391

A significant difference was observed regarding age, surgical duration time, tourniquet time, touch recovery time, and motor block onset time between both groups (Table 1 and Table 2).

In addition, no significant differences in postoperative complications were observed between the two groups (Table 3).

**Table 3:** The frequency of postoperative complications among the studied groups n (%): Number of patients with the complication (n) and percentage relative to the group (%); p-value: Statistical measure indicating the significance of differences between groups; ^b^ indicates the p-value is derived from a specific statistical test (chi-square test).

Complications	Controls group n ( % )	Study group n (% )	P-value
Dizziness	12 (44.5%)	15 (55.5%)	0.491^b^
Nausea	11 (61.2%)	7 (38.8%)	0.230^b^
Vomiting	2 (40%)	3 (60%)	1.000^b^

Analgesic need, whether intraoperative or to reduce tourniquet pain among the compared groups, showed a significant difference in which a high percentage of analgesic needs were among those with lidocaine only; however, there was no significant difference in postoperative need for analgesia between the groups (Table 4).

**Table 4:** The frequency of postoperative, intraoperative, and analgesic needs to reduce tourniquet pain among the studied groups ^b ^chi-square test was used

Variables	Yes/No	Case group N (%)	Control group N (%)	P-value^b^
Need postoperative analgesia	Yes	22 (88)	25 (100)	0.074
	No	3 (12%)	0 (0)	
Need intraoperative analgesia	Yes	0 (0)	25 (100)	< 0.001
	No	25 (100)	0 (0)	
Need for analgesia to reduce tourniquet pain	Yes	1 (4)	24 (100)	<0.001
	No	24 (96)	0 (0)	

## Discussion

The results of this study revealed no statistical differences between the two groups regarding gender, body weight, and ASA classification. A significant correlation was, however, noted between the groups regarding age, surgical time, and tourniquet time. Of interest, while no statistically significant differences were found in touch onset time, pinprick onset time, pinprick recovery time, and motor block recovery time (p = 0.537, 0.076, 0.467, 0.391), there were differences in motor block onset time and touch recovery time (p = 0.001, 0.005). Additionally, postoperative complications were not significantly different between groups. Interestingly, intraoperative analgesia requirement was quite heterogeneous (p < 0.001), while postoperative demand for analgesics was determined not to be significantly heterogeneous (p = 0.074). There have been various studies with varying outcomes regarding the analgesic effect of neostigmine on IVRA.

There have been studies regarding the administration of neostigmine as an adjuvant to a number of local anesthetics and with various doses. For instance, Turan et al. found neostigmine in Bier's block with prilocaine to have much lower VAS scores, higher time to first analgesic request, lower total analgesic requirements, lower sensory and motor block onset times, and longer recovery times compared to a control group [18]. Similarly, Marashi et al. found that neostigmine, when used with lidocaine in Bier's block, postponed surgical pain onset significantly [19]. Sethi et al. reported that the addition of neostigmine to lidocaine not only decreases the onset time of anesthesia but also provides postoperative analgesia during IVRA in upper limb surgeries [20]. In the same manner, Kang et al. said that the addition of neostigmine to ropivacaine in IVRA is useful in outpatient arm procedures [21]. Alkan et al. had effective analgesia with epidural 10 μg/kg neostigmine during cesarean section under elective conditions [22]. Harjai et al. also reported benefits of co-administering epidural neostigmine in prolonged duration of analgesia and causing sedation [23]. On the other hand, several studies, such as one by Atia et al. in Egypt, found no analgesic advantage from the addition of 1 mg neostigmine to 0.5% lidocaine in IVRA, nor did this enhance complications [24]. Similarly, Van Elstraete et al. found no clinical benefits of neostigmine when combined with lidocaine in patients undergoing elective ambulatory carpal tunnel release [24].

Other studies have confirmed our experience where neostigmine as an adjuvant to lidocaine offered no analgesic benefits [13, 25]. Singh et al. found dexmedetomidine to be a better adjuvant to intrathecal ropivacaine, with quicker onset and better analgesia, and also found neostigmine at a dose of 50 μg to improve the quality of the block but added side effects [26]. In our study, while neostigmine did not produce major side effects, this could be attributed to the small sample size and lower dosage used. The lack of the anesthetic benefits of neostigmine in our findings could be due to the physiological limitations, e.g., the blood-nerve barrier at the innermost lamella of the perineurium, to prevent the transmission of the neostigmine to the site of action along with the local anesthetic [27, 28]. Unlike local anesthetics like lidocaine, which readily penetrate nerve membranes, neostigmine is a hydrophilic compound that may have limited ability to cross the blood-nerve barrier and reach AChE sites in sufficient concentrations when administered intravenously. Additionally, the confined distribution of the drug within the limb during tourniquet application may further limit its diffusion to the relevant nerve structures. These factors, along with the lack of sustained receptor engagement, may explain the observed lack of postoperative analgesic benefit despite its intraoperative effects on tourniquet-related discomfort.

In our study, the significant reduction in intraoperative analgesic needs suggests that, although neostigmine had no analgesic effect, it may have had some impact on reducing tourniquet pain. However, the absence of statistical differences in postoperative analgesic needs suggests that neostigmine's analgesic effect advantages may not be clinically relevant. Although neostigmine did not significantly improve postoperative analgesic requirements, the reduction in intraoperative analgesic needs suggests it may help reduce tourniquet pain during surgery. However, without an effect on postoperative analgesia, its overall clinical benefit seems limited.

These findings highlight the importance of weighing intraoperative advantages against longer-term patient outcomes when considering neostigmine as an adjuvant. Moreover, decreased reliance on additional analgesics might facilitate smoother anesthetic management and potentially shorten recovery times, thereby enhancing overall patient outcomes. The study has several anticipated limitations. The study may be limited by the number of willing participants, which could influence the statistical power and generalizability of the results. Conducting the study at a single center may restrict the applicability of the results across diverse settings or populations. The follow-up period is relatively short, focusing mainly on immediate and short-term effects, thus possibly missing long-term outcomes or delayed side effects. Variability in surgical techniques and anesthesia administration could introduce inconsistencies affecting the outcomes, and as an open-label or non-blinded study, biases related to patient reporting or outcome assessment. Based on these limitations, certain recommendations could strengthen future research. Larger, multicenter trials with more heterogeneous populations are essential to enhance the generalizability of the findings. Extending follow-up periods will help evaluate the long-term efficacy and safety, including delayed side effects of neostigmine during intravenous regional anesthesia. Standardizing surgical and anesthetic protocols can help reduce variability and better isolate the effects of neostigmine. Exploring different doses and formulations of neostigmine is also advisable to identify optimal dosing with minimal toxicity. Incorporating additional outcomes such as patient satisfaction, functional recovery, and quality of life will provide a more comprehensive understanding of the analgesic benefits. Finally, designing double-blind studies will help minimize bias, ensuring more objective and reliable results.

## Conclusions

In conclusion, we found that the addition of 0.5 mg neostigmine to 3 mg/kg lidocaine in IVRA has no analgesic effect, and there is no increase in the incidence of complications in spite of a significant reduction in analgesic needs for intraoperative and reduced tourniquet pain among the study group. We did not find any evidence that supports the use of neostigmine as an adjuvant to local anesthetic agents in IVRA.

In summary, while neostigmine did not significantly reduce postoperative pain, its beneficial effect in decreasing intraoperative analgesic requirements, likely by reducing tourniquet discomfort, highlights its potential to enhance intraoperative patient comfort. These findings suggest that neostigmine may have a valuable role as an adjuvant in regional anesthesia protocols aimed at improving intraoperative conditions.
